# Managing stress through the Stress Free app: Practices of self-care in digitally mediated spaces

**DOI:** 10.1177/2055207615580741

**Published:** 2015-05-05

**Authors:** Ian Tucker, Lewis Goodings

**Affiliations:** 1 University of East London, UK; 2 University of Roehampton, London, UK

**Keywords:** Stress, qualitative, health, bodies, technology, app, self-care

## Abstract

In this paper we are concerned with the question of *how we feel* when living in concert with multiple technologies. More specifically, we are focused on the influx of digital apps designed to manage psychological wellbeing. We draw on empirical work exploring one such app, Stress Free, and focus on the experiences of stress and technological tools designed to lessen stress. Our concern is with the way that technologies become part of the *experience* of stress as opposed to solely understanding the app as a tool aimed to reduce the occurrence and severity of stress. This involves taking a theoretical journey through philosophies of technology that provide valuable resources for conceptualising the relational characteristics of digitally mediated stress. Our wider interest is to speak to broader concerns with the movement to ‘digital care’ and the implications for how we conceptualise technology, self and care therein.

## Technological practices of care

Telecare has come to represent a range of technological solutions that are designed to reduce healthcare spending, while also aiming to increase feelings of independence for the healthcare user. Telecare is praised for improving access to care in rural and urban locations for people with chronic physical health problems^1^ and the rapid advances in digital tools means that many forms of psychological distress are now being met with technological innovations (e.g. dementia). The shift to developing tools for psychological distress is premised on the same idea as earlier movements in the telecare field – that increased involvement in one’s care will lead to a sense of independence and empowerment, all of which can be managed at a distance. However, critical analysis of the telecare movement (for examples see Cartwright^2^ and Pols^3)^ instils a need to explore the realities of using these technologies and the broader implications of how care works via these technologies, particularly in the shift to developing tools to address psychological issues. Furthermore, it reminds us of the possibilities for new technologies to coerce people into using the technology in ways that reproduce normative forms of independence and actually reduce the chances of a real sense of independence.^4^ Following this critical perspective, a recent special issue of *Science and Technology Studies* utilised the term ‘Patient 2.0’ as a way of conceptually and empirically interrogating the use of technologies in healthcare.^5^ Patient 2.0 explores what kind of relationships between agency, technologies and bodies are made possible, restricted and encouraged through current healthcare technologies. This shows that critical examination of technology requires looking at how we manage our bodies in relation to technology and what forms of bodily knowing are made possible in and through the technology. This paper will focus on how people manage and organise the body in the use of a new app that is designed to deal with stress.

Technologies work to bring forth a form of cultural information that provides a particular way of knowing the body. Technology is constantly producing new resources through which the body can be known. For example, Lopez and Domenech^6^ identify that autonomy in one particular telecare service was governed by the decision to wear a pendant that includes an alarm that can be used to signal the need for help at any time. The decision to engage with the pendant raises a question of the autonomous body, as it is clear that the pendant displaces the body and impacts on the ability to feel autonomous. The authors conclude that ‘the use and implementation of these technologies enacts a fragile and constantly-at-risk body that requires monitoring care and self-surveillance (p.191). The body is ‘constantly-at-risk’ as the presence of the pendant means that the wearer must continue to evaluate if they need medical attention. The body is locked in a constant state of awareness where there is an ongoing need to reflect on whether they need to activate the device. The introduction of a new technology provides a good opportunity to assess the kinds of ways that the body is brought forward.

In this paper we examine how the body is made relevant in the use of a particular technology that is designed to manage, organise and potentially improve psychological wellbeing by reducing stress. The app is called Stress Free (Virtually Free, 15 Warwick Road, Stratford Upon Avon, Warks CV37 6YW) and is one of a number of technologies that focuses on dealing with a psychological form of wellbeing. For instance, computerised cognitive behavioural therapy (cCBT) builds on traditional self-help tools in a way that utilises relational features of an online space.^7^ Other clinical interventions include mood tracking apps, remote access to health services and GPS-based location services (see Luxton et al.^8^ for a useful summary). The shift has led to a new field being designated ‘mobile health’ (mHealth).^9^ Commercial technology companies are also producing digital media tools that target psychological forms of distress, e.g. stress, phobias and mood disorders. The current paper investigates one of these technologies. These interventions are premised on the idea that technologies have the power to intervene in people’s psychological activity. This poses an interesting question in relation to the nature of the relationship between psychology and technology. Are the latter just passive tools to be used by people through pre-planned activity to manage wellbeing or do they have power and agency in and of themselves to affect the human condition?

In this paper we use stress as a condition, and experience – through which we explore questions of agency, bodies and ontology – it as something that can be *felt* in and through practices that are psycho-physiological, and consequently of value in addressing questions of distress, embodiment, agency and technologies. These kinds of questions centre on a concern with understanding how what comes to be seen as actualised experience and activity is contingent on practices that are simultaneously human and technological,^10,11^ and therefore making a theoretical distinction between the two does not help in understanding the reality of social activity emerging in and through embodied-technological assemblages.

Adrian MacKenzie^12^ prefers the term ‘technicity’ to technology as it facilitates a different signification; one that emphasises the relationality of body–technology connections, rather than conceptualise them as fundamentally distinct. Furthermore, the concept of technicity shifts the analytic perspective somewhat, as it defies us to consider the context of an object’s concrete individualisation, which should not be taken as being determined by some form of internal essence or defined set of properties. For MacKenzie,^12^ ‘the technicity of an element is heightened or diminished according to the relative independence it displays in relation to variations in context’ (p.13). As such, technicity directs us towards considering how it is that a given technology (e.g. mobile app) comes to produce a specific actualised effect. In this sense, the whole notion of a stress reducing app can be framed as technicity, in terms of it being designed to have a particular effect on those that use it (i.e. reduce stress). Social scientific analysis of the impacts of such technical elements involve identifying the forms of technicity that specific technologies co-constitute by conceptually mapping the process of technology to technicity.

The shift towards a notion of technicity is useful when thinking about forms of technological care emerging through digital tools to improve psychological wellbeing. For such an intervention to be able to have an effect we have to think of it as intrinsically linked to psychological processes; as interacting directly with the *felt* aspects of experience. We know people are increasingly living with technologies, and whilst a lot of this has been in terms of maintaining relationships through social media as well as a variety of leisure activities (e.g. shopping, listening to music etc.), accessing health services and consequently engaging in practices of ‘digital self-care’ are becoming increasingly prominent.^13^ In this sense we have to *invent* new ways of managing our bodies in and through digital technologies. It is not so much that we are extending our bodies through technologies, but that we are working in patterns of activity that are as much technological as biological.

This paper aims to look at the ways that people use the app Stress Free in terms of how the body can become known within a particular cultural space. In this instance the cultural space is the Stress Free app and the focus is on how the app commands a certain way of feeling about the body as a potentially stressful object. It is important that this form of bodily knowing is shaped in terms of a non-dualistic relationship between the individual and the collective.

## The individual and the collective

With the emergence of psychologically focused digital media we see that such technologies are coming to ‘regulate the existence’ of different forms of psychological distress (e.g. stress and anxiety). For the 20th century philosopher of individuation and technology Gilbert Simondon, there is a tension in the realisation of how individual experience is formed in concert with technologies, in ways that are not entirely pre-figured. Here Simondon draws out a point regarding the way that the event of individualisation (biological and physical) is always produced amidst a broader sphere of potential activity (what he calls the *pre-individual*), and as such, occurs as simultaneously collective and individual:

The subject can be conceived of as the unity of being as an individuated living being, and as a being that represents its actions through the world to itself as an element and as a dimension of the world (Simondon,^14^ p.8).

This sits within Simondon’s broader philosophy that frames the ‘human’ as always-already collective, and given that technologies are agents in the production of collective life, they are by definition, constituents of ‘humanity’. The crucial point for Simondon is to prioritise analysis of individuation over individuality, so that we need ‘*to understand the individual from the perspective of the process of individuation rather than the process of individuation by means of the individual*’ (Simondon,^15^ p.301, original emphasis). Simondon argues that to focus only on individuality as a concept does not incorporate the broader context of the process of individual experience; ‘individuation, moreover, not only brings the individual to light but also the individual-milieu dyad’ (Simondon,^15^ p.301). Consequently, we can think of technologies as engendering processes of individuation, which form as embodied patterns of everyday life. Technologies, although individual in one sense, are also heavily collective in terms of being accessible and offering potentialised activity to societies and culture as a whole. This captures the interest in the current paper about what practices of individuation emerge when using Stress Free.

## Technological affect

Hansen^16^ argues that the issue of experiencing oneself as a body between individualism and preindividualism is felt in moments of *affectivity*. Here, Hansen draws on Simondon’s account of affectivity being the mode in which individual bodies are experienced as somehow incomplete, as needing to be resolved. For Simondon this is a continuous feeling that does not diminish through some form of completion of embodied experience. In this sense the body cannot become ‘whole’, as it is always in a state of ‘making future’. That is, bodies are continuously trying to find the means by which to enter forms of fixity, and yet such endeavours will always fail. The point, for Hansen, is that it is the collective (Simondon’s ‘pre-individual’) that individual bodies have to engage with in their search for completion. This connecting with collectivity, which is a necessary compulsion, is felt as a form of anxiety. Accordingly, bodies can never quite feel at total ease with themselves as a result of this continual need to work with the collective, which of course cannot be *known* in the same way as one’s individual body (as Bergson^17^ noted so well). In this sense we are reliant on the non-human technics that co-constitute the material environments within which everyday life unfolds. This means that concepts that are commonly taken to be uniquely human (e.g. feelings, thought etc.) actually need to be conceptualised as products of the relationships that come to be *between* bodies and technics. Hansen captures this well when stating, ‘the body’s capacity to act is never simply a property it possesses in isolation; it is always a recursive and constantly modulated function of its embeddedness within a rich texture of sensation’ (Hansen,^16^ p.186). Accordingly, we are encouraged not to reduce concepts of feeling (i.e. emotion and affect) to a purely physiological level, as this directs attention away from the other parts of the nexus that constitute their emergence. Instead we are encouraged to follow Bergson’s notion of bodies as ‘centres of indetermination’ (Bergson,^17^ p.35); namely, bodies are always-already incomplete, and are tasked to organise and manage their activities in relation to the ‘excessive realm’ of the technical environments that surround us.

Hansen^16^ invites us to think about the ways that contemporary forms of media allow people to be taken outside of their immediate habitual experience in order to be able to experience something new and engage with ‘something that would not otherwise be experientiable’ (p.223). The use of apps allow people to get a sense of their body in a different way, perhaps as a ‘stressed body’ or a ‘digital body’ or as a ‘relaxed body’, where each of these different versions of the body is embodied in the way that people organise their image of their bodies in the app. Hansen^18^ describes how the ‘digital image’ involves the reappraisal of the relationship between the user’s body and the ‘image’ of the body, where the ‘image’ of the body encompasses ‘the entire process by which information is made perceivable through embodied experience’ (p.9). Here the world is composed of an aggregate of images where the person perceives a subset of this aggregation by isolating certain images that form a ‘centre of indetermination’.

Hansen explains how the body is continually being created anew in the process of filtering information and in new digital media. Digital images of the body have an extended margin of indetermination that give rise to experimentation with different ways of giving life to digital information. Following Bergson, Hansen accepts that the body is a special kind of image that is used to organise all other images and one that is prioritised above all others. So we must accept that when thinking about the role of digital images that these still must be ordered in relation to the ‘privileged’ position of our bodies. Our body is the ‘central organising site’ of our experience and it is through these digitised bodily trials that we are able to get a sense of how our bodies act and feel, resulting in the concept of building an affectual understanding of our own body through the ability to engage and develop our digital image of the body. In this paper we trace a specific example of how the body filters information in one app in order to create an image of the body that gives rise to an affective experience. The booming digital culture of mobile health apps, ranging from those concerned with general wellbeing to specific forms of mental distress, are one part of an individual’s system of apps on a mobile device and are consequently ready-to-hand at any moment.

## Stress Free

In this paper we focus specifically on an app designed to reduce stress. The app is based on a model that utilises a series of behavioural exercises to help users reduce or avoid stress (i.e. calm breathing, deep muscle relaxation, self-hypnosis, meditation). The app is of interest because it is a good example of a first wave of digital media that focuses on psychological issues. Another reason the app is of interest is because we can frame these technologies as networked activities of movement that induce sensations, whether they are directly designed to or not. Furthermore, this app allows for a timely exploration of the wider cultural dynamics that are embedded in this new form of technical care and how these technologies enact reductions in psychological and/or physiological distress.

**Figure 1.**
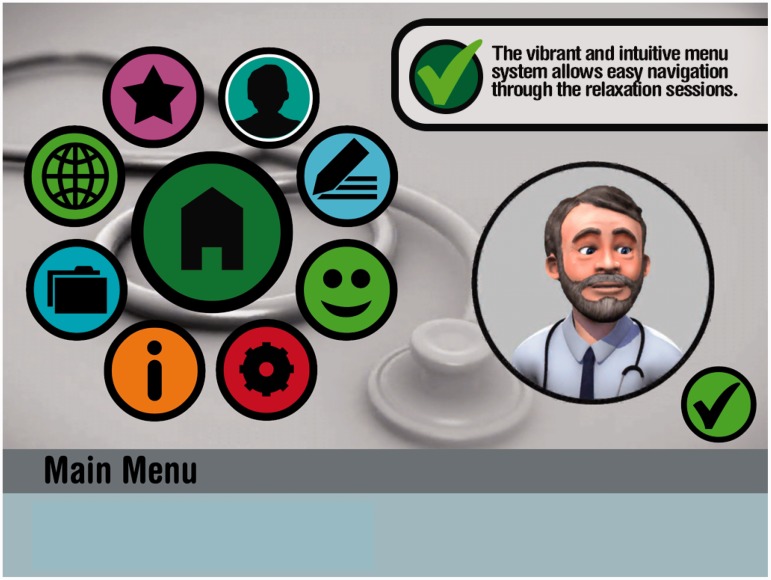
The Stress Free app. Reproduced with permission from Virtually Free.

Figure 1 shows how the app appears on an iPad, with the main menu on the left-hand side of the screen. Users can move their way around the entire app, with each of the icons representing a different activity within the app. On choosing to complete one of the relaxation sessions the user is taken to a similar screen in which each of the icons represents one of the different forms of relaxation. Users are required to work through each of the sessions in turn and in doing so ‘unlock’ future sessions, beginning with calm breathing and developing to deep muscle relaxation. Figure 1 also shows Dr Freeman on the right-hand side of the screen. In each session Dr Freeman explains how to complete each of the relaxation techniques and gives the user time to complete each of the exercises (which is often accompanied by soothing music).

The app also includes an anxiety rating scale that people can use to self-register before and after taking part in an exercise. Users rate their anxiety in the app by selecting a point on a scale between a happy face and a sad face. These ratings are then transformed into a percentage and collected together in a separate part of the app called ‘anxiety statistics’. The app states that users are able to see all of their anxiety ratings across the different exercises and they are able to monitor their stress and anxiety levels. The anxiety statistics function is linked to a journal feature in which users can add content regarding their experiences of taking part in a session or anything else that they may wish to document. There are also auto-entries associated with each session so that users are encouraged to record their feelings after every exercise.

In the next section we will draw upon some empirical research into people’s experiences of using the app Stress Free. At the time of collecting the data for this project Stress Free was relatively new (three months old), and we asked 10 undergraduate students from a London university to use the app for one month and then attend a semi-structured interview to discuss their experience of using the app. Eight females and two males took part. All names have been changed to make them anonymous. The participants were asked to try and use the app once a day but not to feel any commitment to have to use the app, the idea being that it was more likely that they would give an honest appreciation of the app if they had not been specifically told to use the app for a certain number of hours per day. They were also told to work through the app and use as much of it or as little as they wanted. Fortunately, the app is designed in such a way that it is necessary to work through each of the exercises in order to open the new ones. All of the participants worked through the entirety of the content in order to experience all of the exercises.

The analysis focused on the way/s that participants talked about their experiences of Stress Free in a general sense, as well as having a specific focus on the role of bodies and affect in the Stress Free experience. We utilised a discourse analytic approach, in which the interview transcripts were initially coded according to participants’ experiences of Stress Free. This initial stage of coding was followed by a ‘cyclical process’,^19^ which involves shifting between stages of coding and analysis. During this process, coding and analysis are connected processes, with early themes fed back into existing codes, which then inform subsequent analysis. This approach is iterative, and designed to ensure a rigorous and sophisticated analytic approach.

## Analysis

### From stress inducing to stress reducing

One of the first things we noticed was a potential challenge of being presented with a need to re-calibrate one’s view of technology, from something that is often seen as increasing stress to reducing it:

Louise: Before using it I was a bit, I don’t really relate technology with stress free, for me it doesn’t match up particularly because when I think about not being stressed I think about not being near a computer or a phone, I find that more relaxing to just be in like a natural environment. So at first I was a bit unsure and then when I started using it, it’s set out very well it’s very easy to use so that was good, it didn’t stress me out trying to understand how to use it which I thought it might cos some things are just really difficult on iPhones and I don’t get them, but after using it, yeah it’s easy to use and I like the way it’s set out and I like the little music in the background and his voice is quite soothing so, that kind of changed my opinion once I actually started using it.

In this extract we see Louise talk about an initial shift in engagement when she first used Stress Free. Beforehand, technology and its increased presence in life was not something associated with reductions in stress for Louise. Indeed, the idea of reducing stress was firmly entrenched in the notion of moving away from, rather than towards, technology. The anticipation of using the app engenders an initial response that seems to be the opposite of the desired effect of the app – Louise seems stressed out by the use of the app (‘when I think about not being stressed I think about not being near a computer or phone’). The app is designed to move people towards embodied states of relaxation and minimal stress, and yet it is embedded in technologies that can create stress and embody a lack of relaxation for Louise. Noticeably, Louise’s objection to the app is couched in relation to the physical difficulty of being near a computer or phone. These are stressful objects that do not automatically create an opportunity for relaxation. In a general sense there’s a potential stiffening of the body in response to ‘more’ technology and this makes the job of the app more difficult from the start.

Louise describes how these difficulties were met in the app. For example, she describes the particular feel of the app and the ease of use, the ‘soothing’ voice of Dr Freeman (the in-app therapist) and the music in the background. Louise’s narrative demonstrates how the idea of using technology to try to reduce stress has an affective impact on her (‘[the app] changed my opinion once I actually started using it’). She comments on how she now feels positive about using the app to reduce stress after initially thinking that it might be difficult. Her narrative describes an affective transformation that repositions digital technologies as having the capacity to reduce stress in the future. Such transformations, though, often require new patterns of body-technology activities that are not specifically accounted for in the design of Stress Free, as we see in the following section.

### (Re)configuring bodies

With Stress Free we see that reconfiguring existing relationships between bodies and technologies is essential to facilitate care. For example, in the following extract Jacob talks about the way he had to position his body in order to use the app, e.g. lying down.

Interviewer: When you’re doing the techniques, when you’re doing the calm breathing, what do you do with the phone, do you tend to hold it in front of you?

Jacob: That’s quite difficult, because in the beginning, the first time I used it I had to look and it was saying you had to lay down so I was like trying to lay down with my hands held to look at the what the person’s doing and so that was …  because I had to do it again, so that was the bit, I don’t know how to handle that.

Interviewer: It’s interesting isn’t it, because obviously, and I think that’s what I mean about the phone in that, yes we’re now getting relaxing vibes from the phone, but at the same time your phone, holding your phone in front of you isn’t relaxing.

Jacob: No, but I think it takes practice, because the first time when I did it, I used my phone but then the second time I didn’t really have to use my phone I just had to listen to the person, so I put the phone next to me and was just listening to what he said through headphones, so the second time I didn’t need to use my phone, it wasn’t necessary.

The actual direct engagement with the phone or tablet being used required a specific spatial layout so that the screen was visible without needing to hold it. We can see then that a specific set of conditions was necessary before the exercises could be fully engaged with. Jacob had to find a specific body-technology spatial solution to this problem. Initially, holding the phone and using Stress Free did not work, as he could not undertake the bodily relaxation techniques whilst holding the phone. This is an interesting spatial problem. The app wants to help people to relax their bodies, and yet using the app requires work to be carried out on the body that gets in the way of the relaxing body. The transition through these stages, ‘body-using-app’ to ‘relaxed-body’, is not just a straightforward process of using the app. It requires an initial configuration of body and technology, which can then facilitate the work required to achieve the ‘relaxed body’. This demonstrates how use of the app was bound up in a wider set of concerns and challenges, and as such, should not be viewed in isolation as a standalone tool.

Jacob’s comments show how digital apps such as Stress Free are designed to provide technological solutions to psychological issues. The strategies they utilise to do so are entirely bound up in the app itself, i.e. calm breathing and muscle relaxing exercises. What is interesting are the kinds of everyday practices through which the app is put to use. Jacob’s comments demonstrate that, despite the mobile nature of the app, he tended to use the app at specific times and in specific locations, typically in the evening and at home. Moreover, this was not only due to a preference, but some practical issues in terms of the effects of using the app in other locations. For example, the muscle relaxation techniques users found best utilised lying down and many people often wanted to use the app at home rather than in public spaces (e.g. due to the difficulty of undertaking muscle relaxation techniques on public transport). Nevertheless, many of the participants describe a symbiotic relationship with the app where they felt it necessary to keep in close contact with the relaxation exercises.

Mobile apps are designed to be ‘always on’ and ready whenever needed to provide a de-stressing moment. As such, it can be argued that these apps provide a form of care that may have previously taken a different form, be it a chat with a friend, completing a similar set of exercises in an offline environment, or in some cases, it might have been handled by therapeutic interventions. In the following section the participants describe alternatives to the app and we focus on how the participants speak about the differences between the use of the app and traditional notions of care.

### Digital self-care

One of the other issues that arose with using Stress Free was the way it was perceived to internalise stress and the process of stress reduction. The app provides a number of strategies that are designed to provide individuals with strategies to help themselves to combat stress. As we have seen with other digital media initiatives,^13^ a requirement emerges to engage in practices of ‘digital self-care’. The app does not connect users with anyone else, only the virtual Dr Freeman. In the following extract Louise points to how this sits outside traditional strategies for stress reduction, e.g. communicating with others to attempt to talk through underlying stress inducing issues:

Louise: I think it’s because what, I mean for me communication, communicating with someone else, I mean I have, I’ve never been in therapy or anything like that, but I can imagine that it would make me quite relaxed, that it would make me more, I don’t know, I, when I feel stressed I like to talk to people to say this is what’s stressing me out and get advice and get it physically out so I felt that was quite concealing of my stress in my own head and then with my phone rather than being able to really let it out in a kind of physic- if you know what I mean, so I think that’s sometimes why it didn’t always work for me, but then there was also parts of it that were quite, there’s something nice about, you know, being alone, and as I said before the music was quite relaxing and you were in your own zone so that’s why it did work for me, but sometimes, some circumstances where the stress needed to be let go of by really speaking to someone else.

Louise talks about the lack of communication in the app experience. In doing so she draws on the common understanding of therapy being about connecting with another person. The app experience is one in which users can only connect with themselves in the here and now. This means that a sense of movement, of anticipating a potential improvement in the stress situation, is difficult due to the way the app involves repeated engagement with the same strategies. The one part of the app in which a temporal aspect is introduced, the in-app journal, is not really utilised by Louise. In a sense what Louise finds somewhat frustrating is the inability to externalise her stress, to ‘let it out’. We can see how the phone used to access the app is viewed as a kind of extension of the body. To use the phone to engage with in-app exercises is, in a sense, no different to using any other part of the body; the phone is still *her*. At times what Louise needs is someone that is ‘not her’, as she requires something that does not feel like further internalisation of stress. This suggests a limit to the stress reducing possibilities of Stress Free, and potentially to the extent of ‘self-care’ potentialised by the app. This seems to be based on the pre-existing relations between bodies and technologies that the Stress Free activity feeds into. As we saw earlier for Louise, the whole idea of ‘more technology’ is an obstacle to working towards good psychological health through Stress Free. This can be seen to mark a limit to its potential for her, or at least to provide a significant barrier to greater impact. For others, though, their initial patterns of activity could be seen to be more conducive to achieving therapeutic benefits. In the following extract with Robert we see a form of externalisation of stress occur through the app, which acts to set the conditions for later stress reduction:

Robert: I’ve used a journal before, like I had like one on my phone ages ago, think it was called wonderful day or something, and I used to write down my thoughts when I was very anxious and stuff, and I can see, I can definitely see the positives of it, I just never used it, but I can also see the positives of using something like that. You know what I really love though, when you went to the journal thing it showed like your levels so you hadn’t written anything down, you could see the, your stats and if you use that on a continuous basis you can see like a build-up and then there was also like a graph thing I saw which was very good. Those type of things are invaluable, like a very good source of emotion because one it gives you confidence and it also shows that you’re going down the right path. I’ve used a journal before and like the best thing about journals is when you’re in that stressful situation, say if you’ve got like, I like the reminder to use it.

In the above extract Robert talks about the sensation arising when stress is captured in a temporal fashion through the journal aspect of Stress Free. Although he talks about not using the journal to record his feelings, he does appreciate the way the app provides a temporal record of levels of stress across time. This is interesting as the graph seems to work as a way of externalising the stress. At these moments, the stress becomes integrated into the app itself, rather than the app trying to intervene physiologically in the body through the in-app exercises. Robert talks about this techno-temporal record of stress levels becoming a ‘source of emotion’. This resonates with Massumi’s^20^ thinking about the relations between movement and sensation, in that the presence of movement initiates sensory responses. Massumi suggests that the felt sensation of embodied experience is indelibly linked to the movement of relational practices. This is because movement always involves a ‘qualitative difference’, which induces a feelingful response. The sensation that is felt at the level of the individual body then can be understood as fundamentally dependent on the nexus of activity that hinges on the shifting pattern of relational activity dependent on all actors in the nexus. For Robert it is the visualisation of the movement of stress over time that produces an emotional reaction. Moreover, the app can come to play a role in terms of providing a structure to the ongoing stress experience:

Robert: When I, personally when I actually wrote through my anxiety I, the major steps that helped me was going back to the places that I fear, like I felt I couldn’t, but this is cos I had general anxiety disorder so that helps, but also writing things down in journal was able to clear this kind of backlog in my mind and that’s why we have stress, it’s because everything in our minds is resurfacing. I’ve had it like when I’ve read books before, like I’ve had it as like, it’s a seed in your head and your thoughts grow but then you don’t kind of … ,the growth of the seed grows bad so you’ve kind of stopped the seed from growing the correct way and then you become negative and then it kind of builds up you know this kind of negativity it’s hard to, it takes time, it takes effort, that’s why an app like that is very good because one you don’t, you’re not restricted with time, two you can take your time and do it, three you’re told how to do it the correct way which is one of the best things and what’s I really like about the video like I said, prefer sometimes maybe to the subtitles just go through it quicker.

In the above extract the idea of stress being indelibly linked to specific places is presented. Robert talks about how visiting certain places that have previously induced stress and anxiety can be part of the therapeutic journey towards lowering stress levels. The Stress Free app comes to offer an additional opportunity to de-stress through externalising the stress experience. Robert offers a visual representation of the stress in the form of a ‘seed growing bad’. This seed he positions in the ‘mind’, and is something that grows ‘bad’ over time, polluting the body in the form of negative stressful energy and sensation. Using Stress Free for Robert provides a structural intervention for anxiety, a way of channeling the emotion through the pre-existing in-app exercises. At those times the anxiety lessens and seems more manageable. Robert presents Stress Free as a way of ensuring the seed grows ‘normally’, which lessens the chances of stress and anxiety developing.

Robert presents the app experience as enjoyable and stress reducing due to the way that it fits into a journey of lowering anxiety. This has included a stage of place-based activity (visiting places Robert has previously found anxiety-provoking, followed by a journal stage facilitating reflection on the experience of visiting stressful places). The temporal flexibility of this approach is valuable for Robert, and something the Stress Free app facilitates. In this respect the app becomes a self-care tool, which can structure new strategies of stress and anxiety management.

In the above extracts we see how stress is experienced in an ongoing fashion, something needing to be channeled and managed. For Louise, doing this required coming to terms with her affective responses to technologies at a device level. Louise needed to come to see her phone as a tool that could be used to reduce stress through Stress Free, rather than being solely something that tends to increase stress through the ways it connects her with so many things. This level of connection can in fact be stressful, so acted as an initial obstacle to engagement with Stress Free. For Robert, the engagement with Stress Free was more direct. He was able to connect with the app in ways that allowed him to translate non-technological practices into digital form. His previous anxiety and stress had been problematic and through the app he was able to create new practices of managing the stress experience.

## Direct and indirect technogenesis

What we have seen in the analysis are some of the potential issues that can arise when people engage with digital technologies that are designed to intervene in psychological issues where the object of attention is specifically modelled as interior and individual (i.e. personal stress). Apps such as Stress Free are premised on the idea that stress is psycho-physiologically based, and that intervening at the level of the individual is a potentially valuable strategy. At an ontogenetic level technologies can produce an affective response in the form of awareness of recognising that one’s body is always-already organised and managed in a relational nexus with technologies (and other people). This means the actual relation with technologies is affectively registered, and consequently has to be managed as such. This can become a first hurdle (and hence obstacle) to people’s anticipation of any potential benefits of working with digital apps designed to improve psychological health. This level of ontogenetic configuration is not usually factored into the design and implementation of digital apps, which tend to work on the premise that increased digital media use means people are primed to use such technologies in any aspect of their life (e.g. managing health as well as work, social relationships etc.). In a sense, then, the relationship people have with technologies works on several levels. There is an anticipatory relationship at a more general level through which people relate certain technologies as a whole as holding the potential to increase stress. This initial phase can be followed by a more specific relationship with a digital app that can be more stress reducing.

An increasing proportion of our everyday lives is unfolding in close relationships with technologies, and as such we are coming to feel closer to a virtual realm of potentialised activity (which is exactly what Simondon^15^ was focused on with his concept of preindividuality). This relationship, though, is far from straightforward. On the one hand it comes from a desire for the high frequency and multiple connections that digital media make possible. On the other hand, with awareness of an ‘always on’ culture comes increased pressure and awareness of ever-ready catalysts and compulsions for new action. The psychological impact of this is yet to be fully understood, and yet we saw with Louise that it can be stress inducing. Here the stress is not some internal element of Louise’s life, but is fundamentally contingent on the relationship with the app. Whilst the app is designed to lower stress (or avoid its increase) at an individual physiological level, it needs to be understood as residing in pre-existing relationships with technologies that have affective impact. The strategies digital media develop as aids for psychological health need to recognise they are operating within a nexus of pre-existing relationships, which all have affective components.

Hence, understanding and empirically studying the impact of digital media on psychological health and wellbeing is not solely a task of identifying the specific effects of a given technical tool (e.g. Stress Free). What is required is a conceptual approach to identifying how the use of a given tool is situated within a broader set of relations between bodies and technologies, through which individual experience is produced and is played out against. We find of particular use the work of Mark Hansen and Gilbert Simondon, as they offer an understanding of how bodies are ever subject to having to reconfigure in light of new demands from technologies (and yet they remain the primary site of action), as well as a broader body–technology ontogenesis that frames individual activity as always the product of processes of individuation emerging from collective sets of techno-biological activity. In this paper this approach informed analysis of participants’ use of Stress Free and the challenges and benefits experienced. Approaching their use of the app solely in terms of its effects on stress in an individual psycho-physiological way (e.g. by evaluation impact of the app’s relaxation techniques) would miss the broader contextual factors that actually shape the everyday use of the app.

In this sense, adopting a ‘social’ approach to understanding digital media tools designed to aid with psychological issues is valuable. As Simondon^15^ taught us, the very notion of ‘individuation’ needs to be understood as a ‘product’ of wider sets of relations, of which technologies play an indelible role. The ‘digital age’ we are currently living through provides a new theoretical and empirical target for Simondon’s thinking (most of which was focused on pre-digital technologies). The individualistic medical model approaches of the mHealth field are recruiting digital media at an increasingly fast rate. Their internalistic approach to psychological distress fails to incorporate the broader contexts of the *experience* of distress (such as stress and anxiety). To gain analytic insight into the impacts of digital apps such as Stress Free on psychological distress we argue for a social scientific approach that gives equal weighting to bodies and technologies in the production of stress. Moreover, doing so facilitates an important focus on the existing relationships people have with technologies that can be disrupted by apps such as Stress Free, and consequently affect what new patterns emerge.

We have seen that the ways that bodies interact and co-create with technologies is *affective*. So the affective elements at play in mobile app use take place at multiple intersecting levels. This includes the general level of impact of living with technologies such as mobile phones (as we saw with Louise), as well as the effects of using specific apps. The experience of using Stress Free is bound up in a broader affective nexus of pre-existing relations with technologies that are (re)configured through using the app. Consequently, any change to the stress experience brought about by use of the app cannot be entirely reduced to the app itself. What it can do, though, is shift existing patterns of action, and create new body–technology relations through which affective novelty can arise (e.g. the structure Stress Free provided to Robert’s anxiety experience that worked to manage its reduction). Mapping this novelty and (re)configuration of bodies in the ongoing organisation and management of psychological health is an increasingly important task for social scientific analysis.

## References

[1] Demiris G Speedie S Finkelstein S A questionnaire for the assessment of patients’ impressions of the risks and benefits of home telecare. Journal of Telemedicine and Telecare 2000; 6(5): 278–284. 11070589 10.1258/1357633001935914

[2] Cartwright L Reach out and heal someone: Telemedicine and the globalization of health care. Health 2000; 4(3): 347–377.

[3] Pols J Care at a distance: On the closeness of technology 2012; Amsterdam, Amsterdam University Press.

[4] Mort M Roberts C Callén B Ageing with telecare: Care or coercion in austerity? Sociol Health Illnesss 2013; 35(6): 799–812. 10.1111/j.1467-9566.2012.01530.x 23094945

[5] Danholt P Piras EM Storni C Guest editorial: The shaping of patient 2.0: Exploring agencies, technologies and discourses in new healthcare practices. Sci Tech Stud 2013; 26(2): 3–13.

[6] Lopez D Domenech M Embodying autonomy in a home telecare service. In: Latimer J Schillmeier M (eds)Un/knowing bodies 2009; Oxford, Blackwell Publishing,pp. 181–196.

[7] Barazzone N Cavanagh K Richards DA Computerized cognitive behavioural therapy and the therapeutic alliance: A qualitative enquiry. Br J Clin Psychol 2012; 51(4): 396–417. 23078210 10.1111/j.2044-8260.2012.02035.x

[8] Luxton DD McCann RA Bush NE mHealth for mental health: Integrating smartphone technology in behavioural healthcare. Prof Psychol Res Pract 2011; 42(6): 505–512.

[9] Ben-Zeev D Begale M Brenner CJ Development and usability testing of FOCUS: A smartphone system for self-management of schizophrenia. Psychiatr Rehabil J 2013; 36(4): 289–296. 24015913 10.1037/prj0000019 PMC4357360

[10] Latour B Science in action: How to follow scientists and engineers through society 1987; Cambridge, MA, Harvard University Press.

[11] Latour B We have never been modern 1993; Cambridge, MA, Harvard University Press.

[12] MacKenzie A Transductions: Bodies and machines at speed 2002; London, Continuum.

[13] Tucker IM Goodings L Sensing bodies and digitally mediated distress. Senses Soc 2014; 9(1): 55–71.

[14] Simondon G The position of the problem of ontogenesis. Parrhesia 2009; 7: 4–16.

[15] Simondon G The genesis of the individual. In: (Crary J Kwinter S)Zone: Incorporations 1992; Massachusetts, MIT Press, pp. 297–319.

[16] Hansen MBN Bodies in code: Interfaces with digital media 2006; London, Routledge.

[17] Bergson H Matter and memory 1988; New York, Zone Books.

[18] Hansen MBN New philosophy for new media 2004; Cambridge, Massachusetts, MIT Press.

[19] Potter J and Wetherell M. *Discourse and Social Psychology Beyond Attitudes and Behaviour*. London: Sage, 1987.

[20] Massumi B Parables for the virtual: Movement, affect, sensation 2002; Durham, NC, Duke University Press.

